# Prognostic Model for Progradient Tuberculosis Course in HIV-Infected Children

**DOI:** 10.17691/stm2020.12.2.09

**Published:** 2020

**Authors:** S.N. Shugaeva, E.D. Savilov

**Affiliations:** Head of the Department of Phthisiopulmonology, Irkutsk State Medical University, 1 Krasnogo Vosstaniya St., Irkutsk, 664003, Russia; Leading Researcher, Irkutsk State Medical Academy of Postgraduate Education — Branch of the Russian Medical Academy of Continuing Professional Education, 100 Yubileyny Microdistrict, Irkutsk, 664049, Russia;; Professor, Head of the Department of Epidemiology and Microbiology, Irkutsk State Medical Academy of Postgraduate Education — Branch of the Russian Medical Academy of Continuing Professional Education, 100 Yubileyny Microdistrict, Irkutsk, 664049, Russia; Chief Researcher, Scientific Center of Family Health Problems and Human Reproduction, 16 Timiryazeva St., Irkutsk, 664003, Russia

**Keywords:** HIV infection, tuberculosis in children, progressive course of tuberculosis, prognostic model of tuberculosis course

## Abstract

**The aim of the study** was to define independent predictors and create a prognostic model for the progradient course of tuberculosis in HIV-infected children.

**Materials and Methods:**

The prospective observational study of tuberculosis cases of HIV-infected children under 15 years of age has been carried out over 2000–2014 using a continuous sampling method (n=65). Two observation groups were formed: the main group comprising children with a progradient (progressive) type of tuberculosis (n=37) and the comparison group of children with a regradient (regressive) type of the disease (n=28). The logistic regression method was used to create a prognostic model. The quality of model approximation was assessed using maximum likelihood function. Indicators of model goodness of fit are the coefficient of concordance (a permissible level of frequency disagreement is less than 20%) and Hosmer–Lemeshow test.

**Results:**

As a result of a paired comparative analysis based on 17 medico-biological, epidemiological and social signs, 11 statistically different parameters have been distinguished. On their basis, a functional prognostic model has been created including six independent predictors: flaws in children observation in the general medical network (b=23.962), absence of Bacillus Calmette–Guérin vaccination (b=20.404), fatal course of tuberculosis in the human source of infection (b=2.762), tuberculosis identification in children under 3 years of age (b=2.620), absence or low adherence to therapy of the latent tuberculosis infection before tuberculosis detection (b=1.859), marked or severe immunodeficiency (b=1.693). The degrees of the risk factors for the progradient tuberculosis course have been established at the following probability values (decile): at 0.3–0.4 the risk is minimal; at 0.5–1.0 the risk is high; at values of 0–0.2 there is no risk of the disease. Programs for quantitative and qualitative assessment of the risk of progradient tuberculosis course in children with HIV infection have been designed to facilitate the model use.

**Conclusion:**

The presented prognostic model is based on the analysis of the obligatory data in the diagnostic search making its use convenient at any stage of rendering medical aid to HIV-infected children.

## Introduction

The course of tuberculosis in childhood may develop in two opposite directions: it may be regradient or progradient [1]. The regradient variant has a high liability to the regression of pathomorphological changes and clinical recovery even without any treatment. On the territory of Russia, clinical forms of tuberculosis meeting the criteria of this favorable type of development are registered in the majority of cases [2, 3]. The other type of the disease course, progradient, appears as clinical tuberculosis variants not prone to “self-curing”. Without medical intervention and sometimes concurrently with anti-tuberculosis therapy (in immunodeficient states), the disease is progressing resulting in the development of multi-organ, generalized forms or chronic processes threatening the child’s life. Thus, the progradient type of tuberculosis has a high risk of realization in immunosuppression. HIV infection is the main cause of immunosuppression [4, 5]. According to our data, the progradient tuberculosis course in HIV-infected children is registered in more than half the cases and occurs 5.3 times more often than in children with a tuberculosis mono-infection [1].

By the present time, data on separate factors increasing the risk of unfavorable clinical tuberculosis course in co-infected HIV patients have been accumulated: natural progression of HIV infection [6, 7], laboratory signs of deep immunodeficiency [7, 8], adverse social medium [8–10], and so on. Therefore, prognosticating the risk of the progradient tuberculosis development in HIV-infected children based on independent predictors will allow for timely correction of preventive and treatment measures.

**The aim of the study** was to define independent predictors and create a prognostic model of the progradient course of tuberculosis in children with HIV infection.

## Materials and Methods

The prospective observational study of all tuberculosis cases of HIV-infected children has been carried out over the period from 2000 to 2014. The study complies with the Declaration of Helsinki (2013) and was performed following the approval by the Ethical Committee of Irkutsk State Medical Academy of Postgraduate Education. The observation lasted for 350 [312–384] days. Informed written consent to participate in the study was obtained from the children’s legal representatives.

Criteria for inclusion into the study were as follows: age up to 15 years and established diagnoses of HIV infection and tuberculosis; children followed up for less than 60 days (lethal cases were not considered) and those whose parents or guardians refused to participate in the study were excluded. The formed continuous sample of participants (n=65) was divided into two groups in the process of observation by expert judgement: the main group comprising 37 children with the progradient type of tuberculosis and the comparison group of 28 children with the regradient type of the diseases.

The investigation was carried out in two stages. At *the first stage*, parameters with statistically significant differences in the analyzed groups were established. A paired comparative analysis was performed on the basis of 17 signs: seven signs associated with tuberculosis infection (contact with a tuberculosis-infected person; fatal course of tuberculosis in a patient being the source of infection; imperfections of radiological examination of the people surrounding a child; absence or low adherence to the therapy of latent tuberculosis infection before tuberculosis detection; the age under 3 years and tuberculosis manifestation; absence of Bacillus Calmette–Guérin (BCG) vaccination; the post-BCG vaccination scar size less than 3 mm); four signs associated with HIV infection (marked or severe immunodeficiency at the time of tuberculosis detection; natural course of HIV infection prior to the disease identification; viremia with a viral load of more than 100,000 copies/ ml at the time of tuberculosis detection; absence or incomplete scheme of preventing the mother-to-child HIV transmission); five social signs (alcohol and/ or drug addiction of adult members of the family; low financial security of the family when income per a family member is less or equal to the subsistence level; parents or guardians without a steady job; one-parent nuclear family; orphanhood including social one); one sign defining the quality of medical aid (flaws of medical follow-up in the general medical network: non-observance of the volume and time of anti-tuberculosis measures in district pediatric settings). At *the second stage* of the investigation, independent risk predictors of progradient tuberculosis course were identified and assessed quantitatively and qualitatively by creating a prognostic model using logistic regression method.

**Statistical analysis.** Data were statistically processed using SPSS Statistics 17.0, Microsoft Excel programs installed on Windows 2010 and recommendations given in the appropriate guidelines [11, 12]. The initial data were presented as absolute (n) and relative values (P). When comparing the sign occurrence frequencies, a relative risk (RR) and the confidence intervals (CI 0.95) were calculated. The statistical significance of differences between the signs was evaluated using χ^2^ criterion and its modifications (Yates’s corrections at n<10; two-sided exact Fisher test at n<5). The level of significance for testing the statistical hypotheses (p) was assumed to be 0.05.

The logistic regression method was used to create the prognostic model [11, 12]. The probability of occurrence of the event ρ was calculated according to the following formula:

$$\rho =\frac{1}{1+e^{-z}}$$

where *e* is a mathematical constant (*e*=2.71828); *z*=β_1_·*x*_1_+β_2_·*x*_2_+β*_n_*·*x_n_*+α; *х*_1, 2, …, *n*_ is an independent variable (0 no sign, 1 presence of the sign), β_1, 2, ..., *n*_ are coefficients of regression; α is a constant.

If ρ is greater or equal to 0.5, we initially accept the statement that the event will occur. The function of maximal likelihood was used to assess the quality of approximation of the regression model [11]. The concordance coefficient (the permissible threshold of frequency disagreement was established to be 20%) and Hosmer–Lemeshow test were taken as indicators of the model goodness of fit [11, 12]. The procedure of “sliding examination” was employed for testing the model for its functionability: removing one by one the examined objects from the data set with recalculation of the model parameters and subsequent comparison of the predicted value with the real outcome by counting the percentage of errors [11].

## Results

The intergroup paired comparative analysis carried out at the first stage of the study has found the statistical significance of differences in the occurrence frequency in 11 of 17 analyzed signs with their greater prevalence in the main group (Table 1). The presented parameters served as the basis for creating a mathematical model for prediction of the progradient course (the second stage of investigation). The inclusion of all 11 parameters into the model of logistic regression resulted in the excess of the specified threshold of the concordance coefficient and the model with the complete set of the potential predictors was recognized unsatisfactory.

**Table 1:** Frequency of occurrence of the statistically different signs in HIV-infected children in progradient and regradient types of tuberculosis course (abs. number/%)

Sign	Progredient tuberculosis type (n=37)	Regredient tuberculosis type (n=28)	χ^2^/р	RR (CI 0.95)
Contact with a tuberculosis patient	35/95	14/50	14.76/0.0001	(1.21.9 –2.8)
Fatal source course of infection of tuberculosis in a human	13 of 35/37	1 of 14/7	–/0.042	(1.15.3 –36.1)
Imperfections of people surrounding of radiological a child examination	34/92	18/64	5.96/0.015	(1.11.4 –1.9)
Absence/of latent tuberculosis low adherence infection to therapy	30/81	10/36	12.01/0.0005	(1.32.3 –3.8)
Tuberculosis under 3 years manifestation of age in a child	29/78	8/29	14.16/0.0002	(1.52.7 –5.0)
Absence of BCG vaccination	15/40	3/11	–/0.011	(1.23.8 –11.8)
Marked at the time and of severe tuberculosis immunodeficiency detection	20/54	5/18	7.36/0.007	(1.33.0 –7.1)
Natural tuberculosis course detection of HIV infection before	16/43	3/11	6.66/0.01	(1.34.0 –12.5)
Viral at the load time of of more tuberculosis than 100,000 detection copies/ml	24 of 30/80	6 of 15/40	5.51/0.019	(1.052.0 –3.8)
Flaws in the in general children medical observation network	34/92	14/50	12.39/0.0004	(1.21.8 –2.7)
Alcohol members and/of or the drug family addiction of the adult	29/78	12/43	8.63/0.003	(1.11.8 –2.9)

Then computations were performed using various sets of potential predictors with a successive step-by-step correction of assessment algorithms. After testing the model workability, the final variant of the model included six parameters (Table 2).

**Table 2:** Predictors of progradient tuberculosis course in children with HIV infection and their regression coefficients (β) calculated during model creation

Predictor of progradient tuberculosis course	Coefficient β
Flaws in children observation in the general medical network (*x*_1_)	23.962
Absence of BCG vaccination (*x*_2_)	20.404
Fatal course of tuberculosis in a human source of infection (*x*_3_)	2.762
Age under 3 years at the time of tuberculosis detection (*x*_4_)	2.620
Absence/low adherence to therapy of latent tuberculosis infection (*x*_5_)	1.859
Marked and severe immunodeficiency at the time of tuberculosis detection (*x*_6_)	1.693

The event probability ρ was calculated according to the formula:

$$\rho =\frac{1}{1+e^{-z}}$$

where *z*=23.962·*x*_1_+20.404·*x*_2_+2.762·*x*_3_+22.620·*x*_4_+ +1.859·*x*_5_+1.693·*x*_6_–26.670; β is the coefficient (see Table 2).

The model goodness of fit was evaluated, on the whole, as adequate with a high level of significance (χ^2^=61.667; p=0.0001). The total percentage of model concordance was 90.8% that corresponds to the permissible level of disagreement (9.2%) at the specified threshold of values less than 20%. The result of the Hosmer–Lemeshow test was 1.172; concordance significance 0.997. The share of correct reclassified observations in the main group amounted to 91.9% (34 of 37 observations) that was the main criterion for adoption of this model (given other conditions were observed). A similar value was in the comparison group — 89.3%. The definition of a threshold value for the likelihood of progradient course of tuberculosis in HIV-infected patients has detected a high degree of concordance of the calculated probabilities and real outcomes. A discrepancy was found only at the probability of occurrence of the event 0.3 (30%), i.e. one case in each group.

When evaluating the risk, minimal probabilities of unfavorable development of the events were taken into consideration and the degrees of the risk of progradient tuberculosis course have been established and expressed in the probability values of 0.3 and greater (decile): at 0.3–0.4 the risk is minimal; at 0.5–1.0 the risk is high; at values of 0–0.2 the risk was absent.

For practical application of the mathematical model, programs for quantitative (see the Figure) and qualitative (Table 3) assessment of the risk of progredient tuberculosis course in children with HIV infection have been designed based on the accepted grades of the risk degree.

**Figure:**
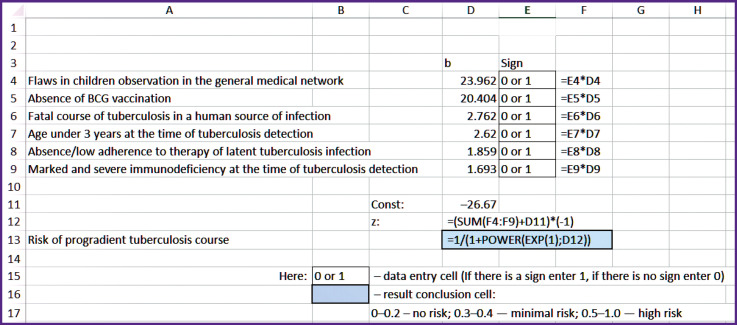
The program of data input (Microsoft Excel) and interpretation of the results of the quantitative risk assessment for the progradient tuberculosis course in HIV-infected children

**Table 3:** Qualitative assessment of the risk degree for the progradient tuberculosis course in HIV-infected children

Number of the identified signs	Risk of progradient tuberculosis course		
	High	Minimal	No
One	—	—	Any sign
Two	**AB** or **AC** or **AD**	**AE** or **AF**	All other combinations
Three	All combinations with **А**	**BCD**	All other combinations
Four	All other combinations except **CDEF**	**CDEF**	—
Five	Any sign combination	—	—
Six	Any sign combination	—	—

Here: **А,** flaws in children observation in the general medical network; **B,** absence of BCG vaccination; **C,** fatal course of tuberculosis in a human source of infection; **D,** child’s age under 3 years; **E,** absence/low adherence to therapy of latent tuberculosis infection; **F,** marked and severe immunodeficiency.

## Discussion

As the result of the mathematical model development, independent predictors have been distinguished among which the parameter “flaws in a child follow-up in the general medical network” was found to have the highest prognostic value: this parameter prevailed among HIV-infected children with the progressive type of tuberculosis (92%; RR=1.8). The influence of this predictor is caused by the untimely diagnosing of the specific process in the district pediatric setting and, respectively, a long period of the natural development of tuberculosis in the HIV-infected child. Additionally, the probability of progressive development of the disease significantly grows due to the predictors characterizing incompetence of anti-tuberculosis immunity: “absence of BCG vaccination” and “marked and severe immunodeficiency” (40%; RR=3.8 and 54%; RR=3.0, respectively).

A household contact with a patient having an active form of tuberculosis is a leading risk factor for being infected in childhood. It should be noted that an extreme manifestation of the epidemiological hazard is the incurable course of tuberculosis in the human source of infection connected, as a rule, with a late diagnosis of generalized, highly contagious forms of this disease. In the medical history of children with progradient type of tuberculosis, this sign (fatal disease course in the human infection source) was encountered 5 times more often than in children with a favorable tuberculosis course (37%; RR=5.3).

An early child’s age was determined by the model as an independent predictor. Liability to disease progression in children in the first three years of life was confirmed by a high occurrence of this parameter with its significant predominance in the main group (78%; RR=2.7). The risk of tuberculosis progression is also aggravated by the absence or inadequacy of chemotherapy effect on the causative agent before the disease development (“absence or low adherence to the therapy of the latent tuberculosis infection”) prevailing among the children cohort with unfavorable tuberculosis course (81%; RR=2.3).

The risk of progradient tuberculosis course using the given parameters can be determined at any stage of rendering medical aid to HIV-infected children. The set of predictors established by us is logically grounded and does not contradict the natural pattern of tuberculosis development in children.

## Conclusion

The presented prognostic model is based on the analysis of the obligatory data in the diagnostic search making its use convenient at any stage of rendering medical aid to HIV-infected children.

Formation of groups with the high risk of unfavorable development of tuberculosis allows for optimization of preventive measures and strengthening of epidemiological surveillance of this category of patients. In case of the tuberculosis process manifestation, a high probability of progradient variant of the disease development together with other criteria can be the basis for correcting the chemotherapy regimen.
